# A New Generation of T7 RNA Polymerase-Independent Inducible Expression Plasmids for *Trypanosoma brucei*

**DOI:** 10.1371/journal.pone.0035167

**Published:** 2012-04-12

**Authors:** Jack Sunter, Bill Wickstead, Keith Gull, Mark Carrington

**Affiliations:** 1 Department of Biochemistry, University of Cambridge, Cambridge, United Kingdom; 2 Centre for Genetics and Genomics, University of Nottingham, Nottingham, United Kingdom; 3 Sir William Dunn School of Pathology, University of Oxford, Oxford, United Kingdom; Louisiana State University Health Sciences Center, United States of America

## Abstract

Expression of transgenes is central to forward and reverse genetic analysis in *Trypanosoma brucei*. The inducible expression of transgenes in trypanosomes is based on the tetracycline repressor binding to a tetracycline operator to prevent transcription in the absence of tetracycline. The same inducible system is used to produce double-stranded RNA for RNAi knockdown of target genes. This study describes a new plasmid pSPR2.1 that drives consistent high-level expression of tetracycline repressor in procyclic form trypanosomes. A complementary expression plasmid, p3227, was constructed. The major difference between this and current plasmids is the separation of the inducible transgene and selectable marker promoters by the plasmid backbone. The plasmid p3227 was able to support inducible expression in cell lines containing pSPR2.1 as well as the established Lister 427 29-13 cell line. p3666, a derivative of p3227, was made for inducible expression of stem loop RNAi constructs and was effective for knockdown of *DRBD3*, which had proved problematic using existing RNAi plasmids with head-to-head promoters. The plasmid system was also able to support inducible transgene expression and *DRBD3* RNAi knockdown in bloodstream form cells expressing tetracycline repressor from an integrated copy of the plasmid pHD1313.

## Introduction

The expression of transgenes is central to many investigations of gene function. Evidence for function can be gained from an investigation of phenotype after expression of transgene at altered levels or in cell types in which there is normally no expression. The transgene can encode the wild type or an altered protein; for example it may be expressed as a fusion with a fluorescent protein to investigate sub-cellular localisation or with a tag to enable rapid identification of interacting proteins. The use of transgenes encoding mutants that are inactive and have a dominant phenotype can be particularly informative. The expression of the transgene can be constitutive or conditional; the latter is essential if expression of the transgene causes a cessation of proliferation. The use of transgenes in functional genomics is the basis of reverse genetics. A second important use of transgenes is in the production of reporter cell lines for forward genetic screens, an approach that is best developed in yeast genetics. For example, a cell line that expresses a fluorescent marker protein can be used in a forward screen for loss of expression and isolated using fluorescence activated cell sorting.

Here, a system for tetracycline-inducible transgene expression in *Trypanosoma brucei* that is independent of T7 RNA polymerase (T7 RNAP) is described. It is a two-component system: a novel plasmid for tetracycline repressor (TetR) expression combined with plasmids for transgene or stem loop RNA expression. The system shows little clonal variation and can be readily introduced into a range of cell lines.

A range of plasmids is already available for both constitutive and inducible expression of transgenes in trypanosomes. The inducible expression systems invariably use the tetracycline system. The TetR binds to tetracycline operator (tetO) sites located within a promoter and prevents transcription; addition of exogenous tetracycline causes release of TetR and permits transcription. The EP1 procyclin promoter was the first to be modified to be tetracycline-inducible [1] [2] and expression levels were titred using a range of tetracycline concentrations. Subsequently the rRNA promoter was modified to be tetracycline responsive [3].

In trypanosomes, mRNAs are processed by the *trans*-splicing of a short exon, called the spliced leader, to the 5′ end. Consequently, it has been possible to achieve high levels of transcription using T7 RNA polymerase (RNAP): the addition of the spliced leader results in a mature capped mRNA [4]. Plasmid systems for tetracycline-inducible T7 RNAP driven expression of transgenes were amongst the first to be developed [5] and the same promoters have been used in head to head RNAi plasmids [6].

In an early set of plasmids exploiting tetracycline-inducible promoters, the selectable marker gene was placed under the control of a constitutive promoter downstream of the inducible transgene, for example pHD437 and derivatives [7] (http://www.zmbh.uni-heidelberg.de/clayton/vectors.html); this arrangement was successful but the degree of expression in the absence of tetracycline was variable [7].

In the second generation plasmids, for example pLEW100 and derivatives [2] (http://tryps.rockefeller.edu/trypsru2_plasmids.html), the tetracycline-inducible EP1 promoter was used for the transgene and a T7 promoter was used for expression of the selectable marker. The two promoters, for transgene and selectable marker, were placed back-to-back adjacent to each other. The choice of a T7 promoter required the background cell line to express T7 RNAP in addition to TetR. However, the use of the T7 promoter had one important advantage, namely that as transcription relies solely on the recognition of the 17 bp promoter by the T7 RNAP, no transcription factors are required. Thus, the footprint of the promoter on the chromatin is small and unlikely to overlap with the footprint of the adjacent transgene promoter. At the time, a second reason cited was that the strong transcription from the T7 promoter ensured that the selectable marker gene was expressed at high levels [2]. Low expression of the selectable marker had been perceived as a possible problem with the first generation vectors [7]. pLEW100 and its derivatives are successful, widely used and available in many flavours [8] (http://tryps.rockefeller.edu/trypsru2_plasmids.html).

The pLEW100 derivatives described above were all targeted to the non-transcribed spacer within the repeat that contains the rRNA genes. There are multiple rRNA loci located on several chromosomes in the *T. brucei* genome and there is evidence that there is variability in background expression and inducibility in different rRNA loci [7] [3]. This problem has been resolved by placing a plasmid targeting sequence containing an incomplete selectable marker gene in one selected rRNA locus [3]. The construct containing the tetracycline-inducible transgene contains a sequence sufficient to reconstitute the selectable marker gene and thus ensures that integration occurs in the desired site.

Subsequent plasmids moved away from dependence on T7 RNAP by replacing the T7 promoter with an rRNA promoter [8] thus removing the need to use a cell line expressing T7 RNAP. The arrangement was similar to pLEW100 with adjacent back-to-back promoters, a tetracycline-inducible transgene promoter and an rRNA promoter driving expression of the selectable marker. These vectors, the pDEX377 series, were designed to integrate in the 177 bp repeats located on minichromosomes. This location was chosen as it results in a lower level of background expression of the transgene in the absence of tetracycline [9]. However, over a couple of years of using pDEX377 derivatives to express transgenes encoding proteins fused to various fluorescent proteins, it is emerging that cells in clonal populations are prone to unreliability, in that expression was markedly variable from one cell to another (data showing this variability is included in the results below).

All the plasmids described above require trypanosome cell lines that express TetR and most also require T7 RNAP expression. The most commonly used procyclic cell line, Lister 427 29-13, contains two integrated plasmids: pLEW13 directing expression of T7 RNAP and TetR using endogenous transcription within the tubulin locus, and pLEW29 integrated into the RNA polymerase I locus and directing TetR expression from a 10% activity T7 promoter [2]. There are several bloodstream form cell lines in common use: Lister 427 13-90 contains integrated copies of pLEW13 described above and pLEW90 in the tubulin locus, directing TetR expression from a 10% activity T7 promoter [2]. Lister 427 ‘single marker line’ contains both T7 RNAP and TetR genes integrated into the tubulin locus: the T7 RNAP is transcribed by the endogenous polymerase and TetR by expression from a 10% activity T7 promoter [2]. Lister 427 1313-1333 contains an integrated copy of pHD1313 placing two TetR genes into the tubulin locus that are expressed through endogenous transcription, and pHD1333 placing a T7 RNAP gene under the control of a tetracycline-regulated promoter in the non-transcribed spacer of the rRNA gene locus [10].

The expression system described here provides an alternative approach to those above. The system has two advantages: it is independent of T7 RNAP and can be readily introduced into existing procyclic form cell lines. Integration of the plasmid pSPR2.1 results in TetR expression with little clonal variation in levels. The remaining plasmids, p3227 for transgene expression and p3666 for double stranded RNA expression, use the tetracycline-inducible EP1 procyclin promoter for regulated expression and an rRNA promoter for selectable marker gene expression.

## Results

### pSPR2.1, a plasmid for TetR expression in procyclic cell lines

**Figure 1:**
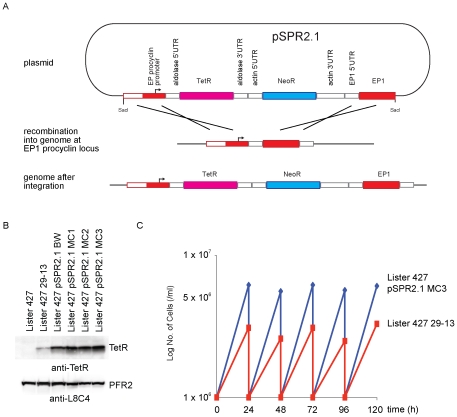
Comparison of the Lister 427 pSPR2.1 and Lister 427 29-13 cell lines. (A) Diagram showing pSPR2.1 and its integration after digestion with SacI. The plasmid integrates between the EP1-1 ORF and promoter. The protein coding regions are represented by the large rectangles. The intergenic regions are represented by the small rectangles and the origins of these regions are labelled above them. (B) Western blot probed with anti-TetR and anti-PFR (loading control) showing the levels of TetR expression in wild type, 29-13, and four independent SPR2.1 cell lines. (C) Comparison of growth of an SPR2.1 cell line and 29-13 cell line.

pSPR2.1 was designed for use with procyclic forms and was constructed to integrate into the *EP1-1* procyclin locus so that *TetR* is transcribed by RNA polymerase I from the EP1 promoter (Figure 1A). The expression of TetR in Lister 427 29-13 (abbreviated to 29-13 cell line) was compared with four independent clones of the Lister 427 after integration of pSPR2.1 (abbreviated to SPR2.1 cell line) (Figure 1B). The expression of TetR protein was significantly higher in all four clones of the SPR2.1 cell lines than in the 29-13 cell line and the expression in four clonal SPR2.1 cell lines was similar. This consistency in expression levels means that pSPR2.1 can be used to modify existing cell lines to obtain reliable expression of TetR.

One SPR2.1 cell line, clone MC3 (Figure 1B), was used for all experiments from this point onwards. There was no obvious morphological phenotype arising from integration of pSPR2.1 and the growth was identical to the parental line (data not shown). The growth of the SRP2.1 cell line was significantly faster than the 29-13 cell line, with a doubling time of ∼9 hours compared to ∼15 hours, but this difference is also present in the two parental cell lines which have diverged over time in culture (Figure 1C).

### p3227, a base plasmid for tetracycline inducible expression

**Figure 2:**
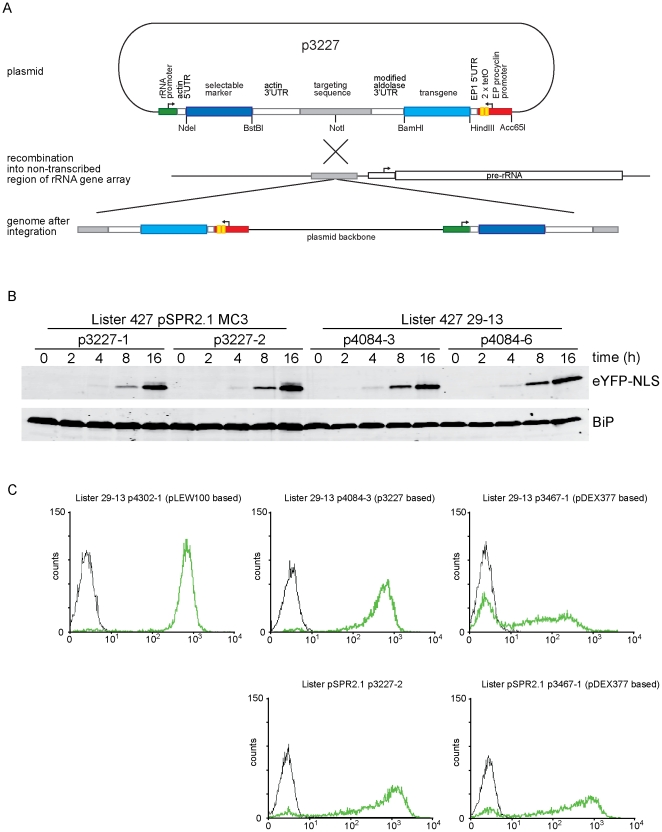
p3227 supports tetracycline inducible expression. (A) Diagram of p3227 and its integration into the non-transcribed spacer in a tandem array of rRNA genes after digestion with NotI. The other unique restriction sites shown allow the replacement of the promoter, transgene and selectable marker. (B) Western blot probed with anti-GFP and anti-BiP (loading control) showing eYFP-NLS expression during a time course after induction in SPR2.1 and 29-13 cell lines. (C) Flow cytometry measurement of eYFP-NLS levels in individual cells from representative clones in which the different plasmids were induced with tet (green line) or without (black line) for 18 hours.

The objective was to make a T7 RNAP independent plasmid for reliable inducible transgene expression without the tendency towards cellular heterogeneity in expression levels displayed by pDEX377. In p3227, the approach taken was to separate the two promoters by locating the plasmid backbone between the tetracycline-inducible transgene and selectable marker gene (Figure 2A). In p3227, transgenes can be exchanged as HindIII BamHI fragments, or using other restriction enzymes to produce compatible ends. The selectable marker gene can be exchanged as a NdeI BstBI fragment and the transgene promoter as a Acc65I HindIII fragment (Figure 2A). In addition, p3227 can be modified to express C-terminal fusion proteins using the same approach as previously described [8].

p3227 is targeted to the non-transcribed spacer in the rRNA locus after linearisation with NotI. Prior to choosing this site, initial experiments had tested integration of a similar plasmid into minichrosome 177 bp repeats, as used with pDEX377 and derivatives [8]. However, unlike pDEX377, no transgene expression was detected in the presence of tetracycline. It was reasoned that the absence of expression might result from chromatin silencing of the inducible promoter as the distance to the active promoter driving the selectable marker gene might be large enough to permit the default silenced state. Two derivatives of pDEX377 were made to test this idea. pDEX377 contains a back-to-back arrangement of the tetracycline-inducible EP1 promoter and an rRNA promoter driving the selectable marker gene [8]; in the two derivatives the promoters were separated by a 250 bp or 500 bp. The expression of an *eYFP-NLS* transgene was determined in two independent clones of each of the plasmids in the SPR2.1 cell line (Figure S1). The expression of eYFP-NLS decreased as the size of the spacer increased, implying that the two promoters need to be very close to each other to achieve maximal expression when the plasmid is targeted to the 177 bp repeats.

The original aim of this work was to produce a T7 RNAP independent expression system and as a consequence a T7 promoter was left in the base plasmid from which p3227 was derived. To test p3227, expression of an *eYFP-NLS* transgene was compared with existing expression plasmids and cell lines and to enable this experiment the T7 promoter was deleted as a 30 bp fragment from p3227 to make p4084; the plasmids are otherwise identical. Two independent clones of each of the cell lines SPR2.1 p3227 and 29-13 p4084 were analysed for eYFP-NLS expression using Western blotting with anti-GFP (Figure 2B). After the addition of tetracycline, all four clones had similar kinetics of transgene expression, with the eYFP-NLS detected after 4 hours, and after 16 hours all clones expressed similar levels. The induction characteristics of the SPR2.1 and 29-13 cell lines were similar and p3227/p4084 was able to support inducible expression in both cell lines.

p3227 was modified to express transgenes with a tag at the C-terminus by adding a BglII BamHI fragment encoding different fluorescent protein ORFs to the BamHI site [8]. p3227 was further modified by the addition of an XhoI site adjacent to the HindIII site making p3927. XhoI HindIII fragments encoding different fluorescent protein ORFs were then inserted between the XhoI HindIII sites in order to express N-terminal fusion proteins. This system of vector modification is the same as used with pLEW100 and pDEX377 earlier [8]. The N and C-terminal tagging plasmids constructed so far are listed in Table S1.

### Transgene expression at the single cell level

The intercellular variability in expression of an *eYFP-NLS* transgene was compared between the plasmids p4084 (a derivative of p3227), p4302 (a derivative of pLEW100) and p3467 (a derivative of pDEX377). Each plasmid was integrated into the 29-13 cell line and p4084 and p3467 were also integrated into the SPR2.1 cell line. p4302 could not be used with the SPR2.1 cell line as it requires T7 RNAP. Two independent clones of each cell line and plasmid combination were analysed by flow cytometry 16 hours after tetracycline induction (Figure 2C). A ∼350 fold induction of transgene expression from the pLEW100 based plasmid in the 29-13 cells was observed; moreover, expression was relatively uniform and there were only a few cells with an intermediate level of eYFP-NLS. In contrast, when expression was induced from the pDEX377 based plasmid in 29-13 cells a ∼100 fold induction was seen with a substantial proportion of cells not fluorescent or expressing an intermediate level of eYFP-NLS. For the p3227 based plasmid in 29-13 cells, there was ∼250 fold induction of transgene expression with substantially smaller number of cells expressing an intermediate level of fluorescence than compared to the pDEX377 based plasmid.

The variability in expression of eYFP-NLS was compared between the p3227 and p3467 in the SPR2.1 cell line (Figure 2C). A ∼350 fold induction of transgene expression was observed from p3227, whereas p3467 only achieved ∼150 fold induction with a greater number of cells not expressing the inducible transgene at all. A higher maximal expression level of eYFP-NLS was observed in the SPR2.1 cells than the 29-13 cells; however, there was a greater range of expression levels.

### Plasmid for inducible stem loop expression for RNAi

A perceived problem with inducible RNAi vectors is incomplete repression in the absence of tetracycline. An initial experiment was performed to determine whether additional tetO sites would reduce the levels of transgene expression in the absence of tetracycline. p3227 was modified and two additional tetO sites were introduced to make p3383. A comparison of the plasmids p3227 and p3383 was performed by measuring eYFP-NLS expression using flow cytometry in two independent clones of each plasmid in the SPR2.1 cell line (Figure S2). The addition of the extra tetO sites did not affect the level of eYFP-NLS present without induction within the detection limit of the flow cytometer. Importantly, the extra tetO sites did not affect expression levels of the transgene after induction.

**Figure 3:**
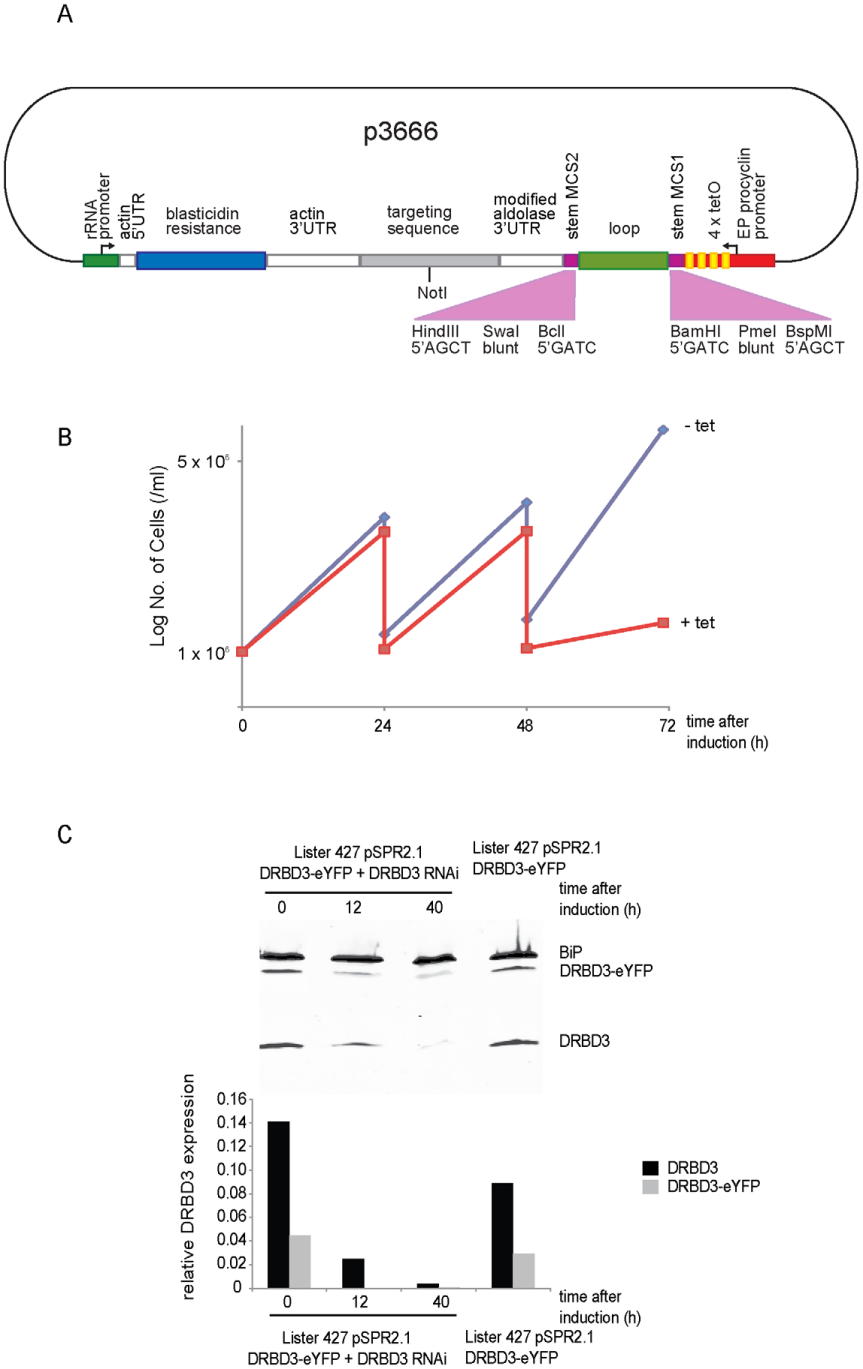
Effective reduction of DRBD3 expression by RNAi based on p3666. (A) Diagram of p3666, the integration into the genome after NotI digestion is the same as shown for p3227 in Fig. 2A. The arrangement of the restriction sites within the stem multiple cloning sites (MCS) is shown. (B) Growth of cells expressing DRBD3-eYFP with or without tetracycline addition. Experiment was repeated with three clones and a representative growth curve is shown. Red line is with tet induction; blue is without tet induction. (C) Western blot probed with anti-DRBD3 of samples collected over a time course after induction of RNAi. The levels of DRBD3 and DRBD3-eYFP expression were normalised against the BiP loading control. Experiment was repeated with three clones and a representative Western blot and quantitation is shown.

p3383 was used to construct p3666, which was designed to express an RNA stem loop on tetracycline induction (Figure 3A). Two changes were made to p3383: first the EP1 5′ UTR, including the splice acceptor site, was removed so *trans*-splicing should not occur. Second, two stem multiple cloning sites separated by a loop sequence replaced the transgene. The stem multiple cloning sites (MCS) were designed for directional cloning of fragments derived from the standard HindIII and BamHI compatible fragments used in the expression vectors described here and previously [8]. Both MCS can accommodate HindIII compatible to blunt, blunt to BamHI compatible or HindIII compatible to BamHI compatible fragments so that they have opposite orientations and produce a stem loop on expression. This design permits the same target gene derived restriction enzyme fragment to be used in two successive rounds of subcloning to produce a stem loop RNAi plasmid.

### RNAi against *DRBD3*

To test the efficacy of p3666 and the SPR2.1 cell line, a knockdown of DRBD3 was performed. The phenotype of the knockdown in procyclic forms has been well characterised and is lethal [11]. *DRBD3* was chosen, as it had previously proved problematic to produce a stable cell line in 29-13 cells using an RNAi plasmid against *DRBD3* based on the p2T7-177 plasmid [9].

The stem loop *DRBD3* RNAi plasmid was integrated into the SPR2.1 cell line containing one wild type *DRBD3* allele and one allele modified to express DRBD3 with an eYFP tag at the C-terminus. Three clones were selected and analysed. The doubling time of the three clones were:12.3 h, 11.9 h, and 10.9 h, this was significantly longer than the parental SPR2.1 cell line at 9.3 h (Figure 1C). The difference could have arisen from one or more of the following: (i) the presence of the RNAi construct, (ii) the presence of blasticidin, (iii) low level RNAi depletion of DRBD3 due to incomplete repression of the RNAi construct or traces of tetracycline in the foetal bovine serum. It is not readily possible to distinguish these possibilities but it is worth noting that there was no depletion of DRBD3 protein detected prior to addition of doxycycline in the SPR2.1 cell line (Figure 3C).

The results of tetracycline addition for one clone, which were typical, are shown in Figure 3B and 3C. Induction of the RNAi caused a large reduction in growth rate after 48 hours (Figure 3B). The expression of DRBD3 and DRBD3-eYFP was analysed during the time course by Western blotting using an antiserum raised against DRBD3 (Figure 3C) [11] (a kind gift of Antonio Estévez). There was a reduction in the expression of both DRBD3 and DRBD3-eYFP after 12 hours and at 40 hours the protein was barely detectable. Before induction, the expression of DRBD3 and DRBD3-eYFP in the RNAi cell line was similar to that observed in the untransformed parental cell line, indicating that there was little background transcription from the RNAi plasmid. The new plasmid and cell line enabled an effective RNAi cell line against DRBD3 to be made.

### Use of the new plasmid in bloodstream form cells

**Figure 4:**
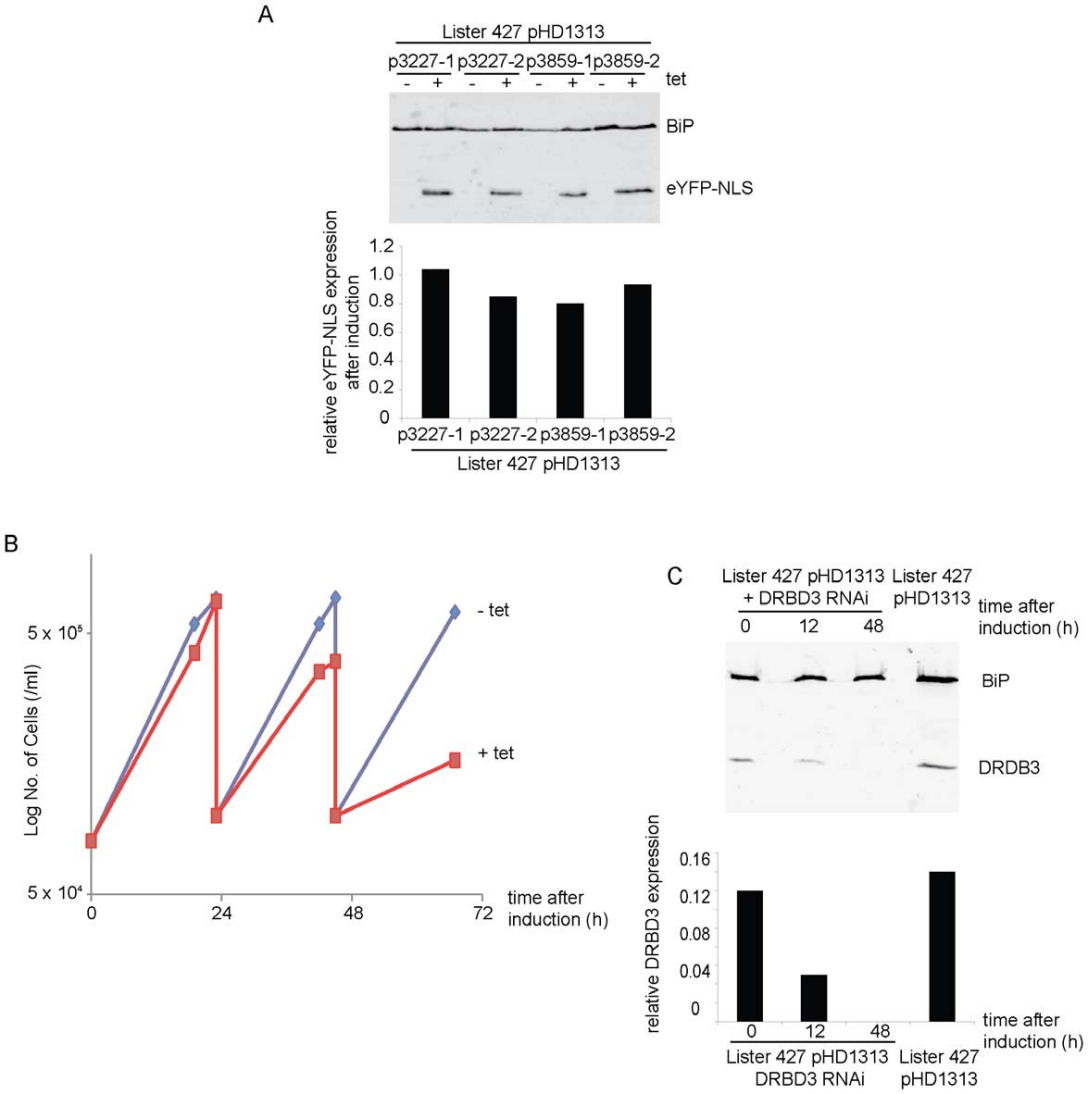
p3666-based RNAi in bloodstream form cells. (A) Western blot probed with anti-GFP showing levels of eYFP-NLS expression in cell lines using either the EP promoter or the rRNA promoter. The expression of eYFP-NLS was normalised against the BiP loading control. Data for two independent clones is shown. (B) Growth of DRBD3 RNAi cell line with or without tetracycline induction. Experiment was repeated with three clones and a representative growth curve is shown. Red line is with induction; blue is without induction. (C) Western blot probed with anti-DRBD3 of samples collected over a time course after addition of tetracycline. The levels of DRBD3 expression were normalised against the BiP loading control. Experiment was repeated with three clones and a representative Western blot and quantitation is shown.

The success of the *DRBD3* RNAi led to a trial in bloodstream form cells. However, pSPR2.1 was not used as it relies on transcription from the endogenous procyclic form specific EP1 locus. To circumvent this problem, Lister 427 bloodstream form (BSF) cells were modified by the insertion of pHD1313 [10] (a kind gift of Christine Clayton) which directs the expression of TetR after integration into the tubulin locus. A second potential problem was the use of an EP1 procyclin promoter in BSFs and a comparison of the EP1 promoter and an rRNA promoter was performed. p3859 was made by modifying p3227 by replacing the EP1 promoter upstream of the tetO sites with the equivalent region from an rRNA promoter. The plasmids p3227 and p3859 were integrated into the BSF pHD1313 cell line and two independent clones were selected for each plasmid. The inducible expression in the four clones was analysed by Western blotting after 16 hours of tetracycline induction (Figure 4A). All four clones expressed detectable amounts of eYFP-NLS after tetracycline addition and there was no significant difference in the levels of expression of eYFP-NLS between the EP1 and rRNA promoters. The level of eYFP-NLS expression, normalised against BiP expression, was ∼6 fold lower in BSFs with either promoter than in PCFs.

The DRBD3 stem loop RNAi plasmid was integrated into the BSF pHD1313 cell line. The effect of DRBD3 RNAi has not previously been analysed in BSF cells. Three clones containing the DRBD3 RNAi plasmid were analysed. The results for one clone, which were typical, are shown in Figure 4B and 4C. After 24 hours of tetracycline induction there was a significant reduction in growth rate. The level of DRBD3 was analysed along the induction time course by Western blotting. There was a reduction in DRBD3 expression after 12 hours of induction and DRBD3 was not detectable after 48 hours. These results suggest that the loss of DRBD3 in BSF cells is lethal as it is in PCF cells. The RNAi system effectively knocked down DRBD3 after induction and also the expression level of DRBD3 observed in uninduced DRBD3 RNAi cells was comparable to the expression observed in the parental cell line, suggesting that there was little background transcription from the RNAi plasmid.

## Discussion

A Lister 427 procyclic form cell line was modified by integration of pSPR2.1 into the EP1 procyclin locus to produce the SPR2.1 cell lines. The expression of TetR was higher in SPR2.1 cell lines than in the 29-13 cell line and expression was consistent between clones. The level and consistency of pSRP2.1-directed TetR expression between clones means that the plasmid can be integrated into existing cell lines to give reliable TetR expression. The SPR2.1 cells grew faster than 29-13 cells; this provides advantages such as allowing a more rapid selection of transformed cells. Furthermore, when expressing proteins using a pLEW100 plasmid and 29-13 cells three selectable markers are required [2], whereas using p3227 and SPR2.1 cells only two selectable markers are required, thereby freeing a selectable marker for another use.

When proteins were expressed from pDEX377, variation in levels between individual cells was observed. Such variation in gene expression between cells within a clonal population is a common observation and is the result of a combination of factors. For successful transcription, a complex needs to be assembled on the promoter. When the promoters are adjacent the assembly of one transcription complex could sterically hinder the construction of a second and create a possible source of noise, which may provide an explanation for the variation in eYFP-NLS expression observed from pDEX377. The plasmid p3227 was constructed with the bacterial backbone between the two promoters, which resulted in less variation in expression than pDEX377 but still resulted in greater variation than pLEW100 derivatives. The separation of the promoters did not completely eliminate the intercellular variability in expression, indicating that this effect is not solely due to the close proximity of the two promoters. In contrast, pLEW100 uses a T7 promoter, which is small and only requires T7 RNAP for transcription, possibly reducing any clash between the two promoter complexes, and here pLEW100 had the smallest range of reporter expression level.

When an earlier version of p3227, identical with the exception of the targeting sequences, was integrated into the 177 bp repeats characteristic of minichromosomes there was no expression of the reporter, implying that the inducible promoter had undergone chromatin silencing. The presence of the selective antibiotic in the media requires the continual transcription of the resistance marker, hence maintaining an open chromatin structure around that promoter. When the selectable marker and inducible promoters are adjacent it is possible that the open chromatin structure necessary for transcription of the selectable marker allows transcription from both. Conversely, if the promoters are separated there will be no selection pressure to maintain an open chromatin structure at the inducible promoter. Evidence for this model was provided by the introduction of a 500 bp spacer between the promoters of pDEX377 resulting in a large reduction in the expression of the reporter. The non-transcribed rRNA gene spacer is less repressed than the 177 bp repeats [9] and was therefore able to support inducible expression from p3227. However, changes in chromatin structure between the selectable marker promoter and the inducible promoter may account for the variation in expression observed from p3227.

A *DRBD3* RNAi plasmid derived from p3666 was used with the SPR2.1 cell line. As DRBD3 knockdown is lethal, it gave a read-out of the leakiness of the new RNAi plasmid in the SPR2.1 cell line. Integration of the stem loop RNAi construct targeted against DRBD3 into the SPR2.1 cell line resulted in many clones, suggesting that the higher expression of TetR in SPR2.1 cells was successfully repressing the expression of the RNAi construct. Moreover, prior to the addition of tetracycline, the abundance of DRBD3 in the cell line containing the RNAi plasmid was similar to the untransformed parental cell line. Induction of the RNAi against DRBD3 gave a significant reduction in growth rate after 48 hours, coupled with a reduction in DRBD3 expression, which was undetectable by 40 hours. The combination of the SPR2.1 cell line and a p3666-derived RNAi plasmid allowed the production of a cell line with RNAi targeted against DRBD3 that matched the previously reported phenotype [11], which had been technically problematic when using the 29-13 cell line in combination with the p2T7-177 RNAi plasmid.

The effectiveness of the p3227 plasmid for inducible transgene expression and p3666 for inducible RNAi was examined in BSF cells. In this case, pSPR2.1 was not used to express TetR, as the plasmid integrates into the EP1 locus, which is repressed in BSF cells. The BSF pHD1313 cell line was used to overcome this problem as these cells express TetR from the tubulin locus. The combination of the BSF pHD1313 cell line and p3227 allowed inducible expression of eYFP-NLS and there was no protein detected when the cells were uninduced, indicating that the expression was tightly regulated. It is worth noting that the relative expression of eYFP-NLS was lower in BSF cells than in PCF cells. One potential cause of the difference in expression level between BSF cells and PCF cells is the use of the EP1 promoter, which could lead to a lower expression of protein in the BSF cells. This idea was examined by modifying the p3227 plasmid so the EP1 promoter was replaced with rRNA promoter, which should not be differentially regulated. However, there was no difference observed in the level of eYFP-NLS expression between the EP1 or rRNA promoter.

The expression level of DRBD3 in the uninduced RNAi cell line was similar to the parental cell line, suggesting that there was minimal knock down in the absence of tetracycline. Despite the lower levels of inducible protein expression achieved in BSF cells, the induction of DRBD3 RNAi resulted in a rapid decrease in DRBD3 protein expression with no detectable DRBD3 present after 48 hours. The loss of DRBD3 resulted in a reduction in growth rate. RNAi knockdown of DRBD3 had only been previously analysed in PCF cells; a recent genome-wide RNAi screen has also found a reduction in growth rate associated with DRBD3 knock-down in BSF cells [12]. The new plasmid p3227 and its RNAi derivative are also effective in BSF cells.

Finally there are numerous ribosomal spacer regions, into which p3227 could integrate and these integration sites may result in different levels of transcription [3]. The next step in the development of p3227 will be to ensure that the plasmid is targeted to the same ribosomal locus each time.

## Materials and Methods

### Trypanosomes

The *Trypanosoma brucei* Lister 427 procyclic cells used for production of the SPR2.1 cell line originated from KG's lab [13]. The Lister 427 29-13 cell line was a kind gift of George Cross [2]. *Trypanosoma brucei* Lister 427 MITat 1.5 (118) bloodstream form cells originated in MC's lab [14]. Transgenic trypanosomes were generated using standard procedures. All experiments were performed with logarithmically growing trypanosomes.

### Plasmids and cloning

Details of the plasmids constructed for this study are described in Table S2 and the sequences are in Table S3. All plasmids are available from the authors and the GCK files are available to download from http://web.me.com/mc115/mclab/downloads.html.

### Flow Cytometry

Mid-log phase density cells (5×10^6^ cells/ml) were analysed with and without tetracycline induction using a BD FACScan (BD Biosciences) in the Department of Pathology, University of Cambridge.

### Western blots

Western blots were performed using standard protocols. The origin of the antibodies was: TetR, Clontech; PFR, monoclonal antibody L8C4; GFP, Invitrogen; BiP from Jay Bangs; DRBD3 Antonio Estévez. Detection was either by ECL or using the Odyssey Infrared Imaging System (LI-COR). For quantification, the Odyssey software was used. The background method used was the average of a three pixel width line at the top and bottom of each band subtracted from each pixel within the band. Unequal loading was corrected by reprobing the blots for BiP.

## Supporting Information

Figure S1 **Expression of eYFP-NLS decreases as the space between the promoters increases.** Western blot probed with anti-GFP. The levels of eYFP-NLS expression were normalised against the BiP loading control. Two independent clones of each plasmid were examined and the percentage of cells fluorescent after induction is shown.
(TIF)

Figure S2 **Flow cytometry analysis of Lister 427 pSPR2.1 p3227 and Lister 427 p3383.** Two independent clones of each cell line were analysed with the typical result presented here. Red line untransformed Lister 427 pSPR2.1, black line uninduced, green line 18 hours tet induction.
(TIF)

Table S1 Table describing inducible tagging plasmids produced.
(DOC)

Table S2 Table describing plasmids used in this study. pLEW100 is described in Wirtz et al. [2]. p2948 and pDEX377 are described in Kelly et al. [8].
(DOC)

Table S3 DNA sequences of all the plasmids used in this study.
(TXT)
