# Supplementary figures and images for: A New Generation of T7 RNA Polymerase-Independent Inducible Expression Plasmids for Trypanosoma brucei
Source: PLoS One. 2012 Apr 12;7(4):e35167. doi: 10.1371/journal.pone.0035167 (PMC3325195; doi:10.1371/journal.pone.0035167)

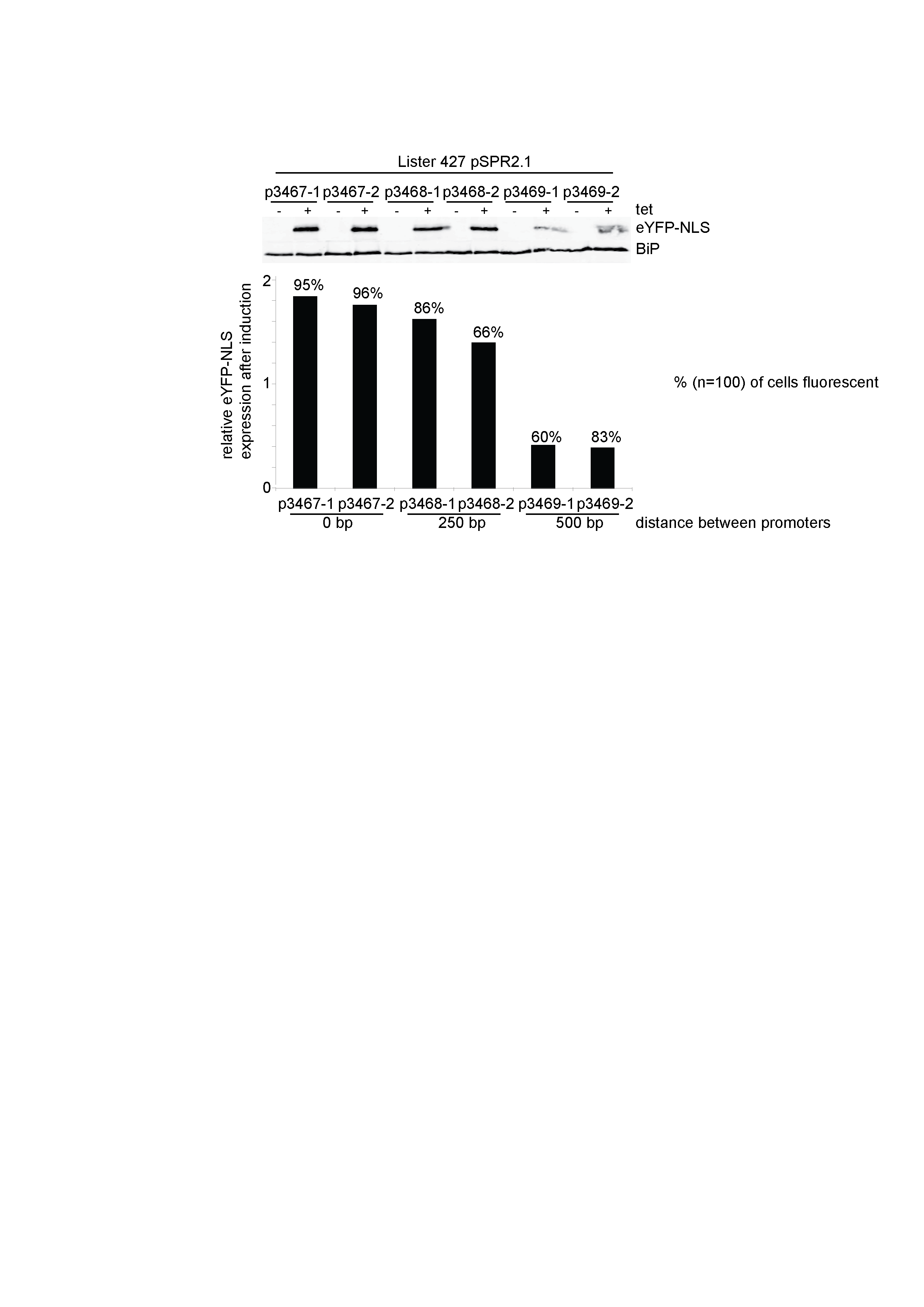

Supplement: Figure S1 — Expression of eYFP-NLS decreases as the space between the promoters increases. Western blot probed with anti-GFP. The levels of eYFP-NLS expression were normalised against the BiP loading control. Two independent clones of each plasmid were examined and the percentage of cells fluorescent after induction is shown. (TIF) [file pone.0035167.s001.tif]

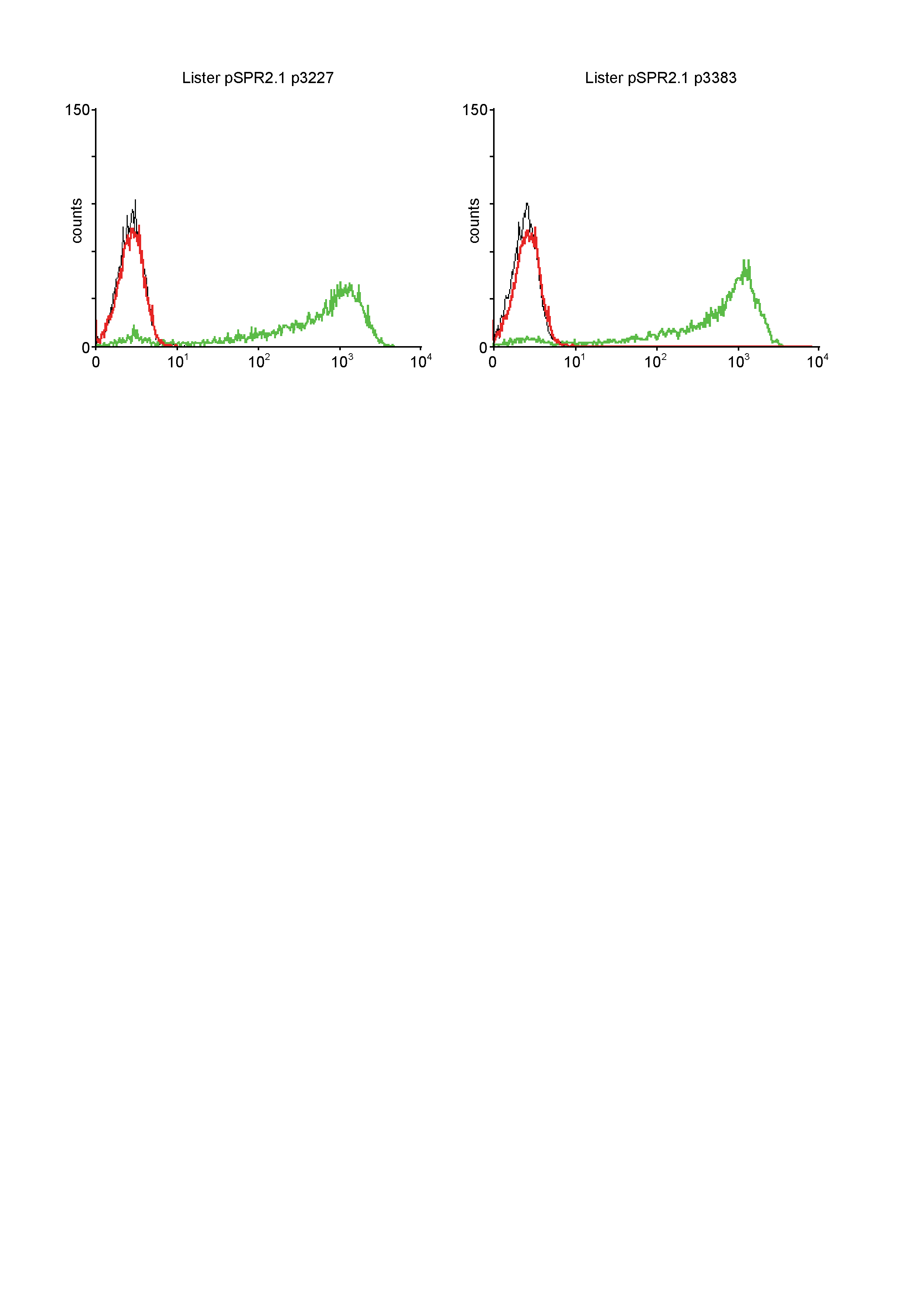

Supplement: Figure S2 — Flow cytometry analysis of Lister 427 pSPR2.1 p3227 and Lister 427 p3383. Two independent clones of each cell line were analysed with the typical result presented here. Red line untransformed Lister 427 pSPR2.1, black line uninduced, green line 18 hours tet induction. (TIF) [file pone.0035167.s002.tif]
