# Supplementary material for: A New Generation of T7 RNA Polymerase-Independent Inducible Expression Plasmids for Trypanosoma brucei
Source: PLoS One. 2012 Apr 12;7(4):e35167. doi: 10.1371/journal.pone.0035167 (PMC3325195; doi:10.1371/journal.pone.0035167)
Supplement: Table S1 — Table describing inducible tagging plasmids produced. (DOC) [file pone.0035167.s003.doc]

| Tag | Plasmids for inducible transgenes | |
| --- | --- | --- |
|  | p3227 N-terminal | p3227 C-terminal |
| eYFP | p3888 | p3944 |
| mCherry | p4006 | p4131 |
| dTomato | p4054 |  |

Table S2

Table describing plasmids constructed for this study. The base plasmid pDEX377 is described in Kelly et al., (2007) and pLEW100 is described in Wirtz et al., (1999).

| Plasmid number | Description | Base plasmid | Resistance marker |
| --- | --- | --- | --- |
| p3227 | inducible eYFP-NLS | n/a | blasticidin |
| p3383 | p3227 with 3 additional tetO sites | p3227 | blasticidin |
| p3467 | pDEX377 with eYFP-NLS | pDEX377 | blasticidin |
| p3468 | pDEX377 with eYFP-NLS and a 250 bp spacer between the promoters | pDEX377 | blasticidin |
| p3469 | pDEX377 with eYFP-NLS and a 500 bp spacer between the promoters | pDEX377 | blasticidin |
| p3665 | DRBD3 hairpin RNAi plasmid | p3666 | blasticidin |
| p3666 | hairpin RNAi plasmid | p3383 | blasticidin |
| p3667 | DRBD3 eYFP C-terminal endogenous tagging plasmid | p2948 |  |
| p3927 | p3383 derivative to produce N-terminal tagging plasmids | p3383 | blasticidin |
| p4084 | p3227 with T7 promoter removed by restriction digest | p3227 | blasticidin |
| p4302 | pLEW100 with eYFP-NLS | pLEW100 | phleomycin |
