# Supplementary material for: A New Generation of T7 RNA Polymerase-Independent Inducible Expression Plasmids for Trypanosoma brucei
Source: PLoS One. 2012 Apr 12;7(4):e35167. doi: 10.1371/journal.pone.0035167 (PMC3325195; doi:10.1371/journal.pone.0035167)
Supplement: Table S2 — Table describing plasmids used in this study. pLEW100 is described in Wirtz et al. [2]. p2948 and pDEX377 are described in Kelly et al. [8]. (DOC) [file pone.0035167.s004.doc]

| Plasmid number | Description | Base plasmid | Resistance marker |
| --- | --- | --- | --- |
| pSPR2.1 | TetR expression plasmid | n/a | G418 |
| p3227 | T7 RNAP independent eYFP-NLS expression plasmid | n/a | blasticidin |
| p3383 | eYFP-NLS expression plasmid with 5 tetO sites | p3227 | blasticidin |
| p3467 | eYFP-NLS expression plasmid with 0 bp spacer | pDEX377 | blasticidin |
| p3468 | eYFP-NLS expression plasmid with 250 bp spacer | pDEX377 | blasticidin |
| p3469 | eYFP-NLS expression plasmid with 500 bp spacer | pDEX377 | blasticidin |
| p3665 | DRBD3 stem loop RNAi plasmid | p3666 | blasticidin |
| p3666 | stem loop RNAi base plasmid | p3383 | blasticidin |
| p3667 | DRBD3 eYFP C-terminal endogenous tagging plasmid | p2948 | hygromycin |
| p3859 | eYFP-NLS expression plasmid with rRNA inducible promoter | p3227 | blasticidin |
| p3927 | expression plasmid with XhoI site inserted upstream of the HindIII site | p3227 | blasticidin |
| p4084 | eYFP-NLS expression plasmid with T7 promoter deleted | p3227 | blasticidin |
| p4302 | eYFP-NLS expression plasmid | pLEW100 | phleomycin |
